# Antibacterial and Anti‐Inflammatory Activity of Chitosan Film with *Rhodomyrtus Tomentosa* Leaf Extract Prepared Via 3D‐Printing Method

**DOI:** 10.1002/open.202400302

**Published:** 2024-11-22

**Authors:** Quan Vo‐An, Chinh Thuy Nguyen, Uyen Thu Pham, Tuan Anh Nguyen, Duong Thanh Nguyen, Dung Tran Hoang, Lu Trong Le, Quyen Thi Cam Ngo, Hoang Thai

**Affiliations:** ^1^ Institute for Tropical Technology Vietnam Academy of Science and Technology 18 Hoang Quoc Viet, Nghia Do, Cau Giay Hanoi 100000 Vietnam; ^2^ Graduate University of Science and Technology Vietnam Academy of Science and Technology 18 Hoang Quoc Viet, Nghia Do, Cau Giay Hanoi 100000 Vietnam; ^3^ University Science and Technology Hanoi Vietnam Academy of Science and Technology 18 Hoang Quoc Viet, Nghia Do, Cau Giay Hanoi 100000 Vietnam; ^4^ Institute of Environmental Sciences Nguyen Tat Thanh University 300A Nguyen Tat Thanh, Ward 13, District 4 Ho Chi Minh City 700000 Vietnam

**Keywords:** Chitosan, *Rhodomyrtus tomentosa*, Leaf extract, Antibacterial activity, Anti-inflammatory, Drug release study

## Abstract

The combination of natural polymers and plant extract attracted much attention thanks to its valuable biological activities. This study presents the preparation and characterization of chitosan (CS) film containing *Rhodomyrtus tomentosa* leaf extract. In which, the *Rhodomyrtus tomentosa* leaf extract (SLE) with polyphenol‐rich fractions was extracted by an ultrasonic‐assisted extraction method. The prepared chitosan/polyphenol‐rich fractions film (CSPPF) was characterized using infrared (IR) spectroscopy, mechanical testing, scanning electron microscopy (SEM), and water absorption analysis. Antibacterial and anti‐inflammatory activities of the CSPPF were assessed using the agar well diffusion method and nitric oxide (NO) inhibition assays, respectively. Besides, the release of the extract from the CSPPF as well as the drug release kinetics was also tested. The *Rhodomyrtus tomentosa* leaf extract enhanced the antibacterial efficacy against *Escherichia coli* and enhanced the anti‐inflammatory activity as well as the swelling ability of the CSPPF. The CSPPF containing 1 wt.% of SLE can inhibit 77.70 % NO with an IC_50_ value of 19.40 μg/mL.

## Graphical abstract

In this study, the chitosan (CS)‐based biocomposite films enriched with *Rhodomyrtus tomentosa* leaf extract (SLE) are prepared by 3D printing method. The CS biocomposite film containing 1 wt.% of SLE exhibits an even structure, adequate swelling degree, and good biocompatibility. This biocomposite film has excellent antibacterial activity against *S. aureus* and *E. coli* and significant anti‐inflammatory effect.

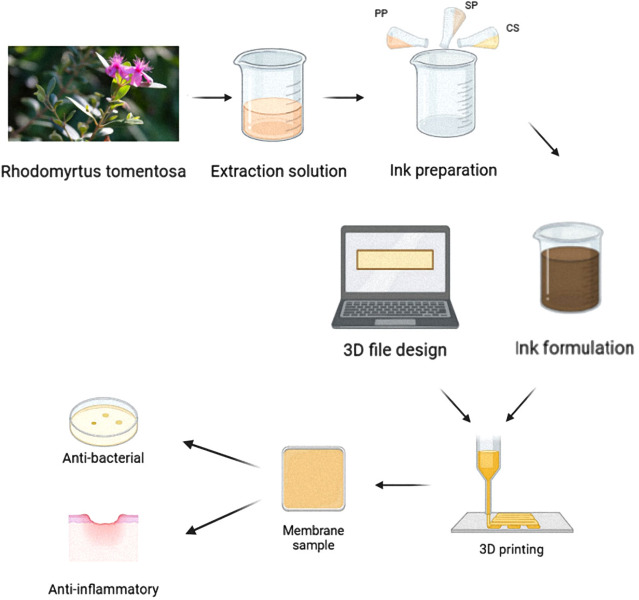

## 1 Introduction

Chitosan (CS), a versatile biopolymer belong to polysaccharide races, has gained prominence as a biomaterial for biomedical applications due to its biocompatibility, biodegradability, and antimicrobial properties.[^1^, ^2^, ^3^, ^4^] It has been applied in various fields of biomedicine, including drug delivery,[^5^, ^6^] antibacterial film,^[7]^ tissue engineering and localized drug delivery,^[8]^ wound healing,^[9]^ cell‐laden.^[10]^ Pure chitosan‐based materials exhibit limitations in mechanical properties and biological activities.[^2^, ^3^]

Other similar natural biopolymers, such as alginate, carrageenan, starch, agar, cellulose, or furcellaran, including chitosan, share similar biocompatibility and biodegradability advantages with chitosan but also exhibit comparable limitations in mechanical strength and functional properties.[^5^, ^6^, ^11^] To address these disadvantages, the incorporating bioactive compounds and plant extracts into biopolymer films, particularly, polysaccharide‐based films have focused in recent research to enhance their functional properties, including antioxidant and antimicrobial activities.^[11]^

While progress has been made in developing films with antimicrobial and antioxidant capabilities, achieving consistent anti‐inflammatory effects remains a challenge. For instance, cottonseed protein hydrolysate‐incorporated alginate films exhibited antimicrobial activity against *Staphylococcus aureus*, *Colletotrichum gloeosporioides*, and *Rhizopus oligosporus*, but not against *Escherichia coli and* lacked anti‐inflammatory effects, as reported by Oliveira Filho *et al*.[^11^, ^12^] Similarly, Koc *et al*. reported enhanced physical, antioxidant, and antimicrobial properties of chitosan films fortified with fungal extracts, surpassing the efficacy of gentamicin, no report of the anti‐inflammatory activity observed.^[13]^ Qin *et al*. focused on the physio‐mechanical properties and antioxidant activity of chitosan films incorporated with montmorillonite and pomegranate rind powder extract. The study also found that the resulting films exhibited antioxidant rather than anti‐inflammatory properties.^[14]^ Roy and Rhim focused primarily on antimicrobial and antioxidant properties when incorporating up to 1 % curcumin into gelatin films with enhanced mechanical properties, antimicrobial efficacy against *E. coli* and *L. monocytogenes*, and antioxidant capacity comparable to ascorbic acid, the anti‐inflammatory effects were not observed.^[15]^

Thus, the existing studies trend in the incorporation of various bioactive compounds into polysaccharide films have primarily focused on antimicrobial and antioxidant properties. Nevertheless, the development of films with consistent anti‐inflammatory effects remains an area for further investigation.

To overcome the limitations of inadequate mechanical properties, various modification techniques for CS have performed, including cross‐linking agents like genipin,[^9^, ^10^] sodium tripolyphosphate,^[5]^ chemical agents^[16]^ or organic compounds.^[6]^ As evidence, the modification of CS with crosslinking agents (sodium tripolyphosphate, sodium citrate, glutaraldehyde) affects the drug release and diffusion of α‐tocopherol from the CS‐based films.^[18]^ The combination of crosslinked chitosan with plant extracts has shown as an efficacy and friendly environmental solution in improving both mechanical properties and antimicrobial activity of CS film.[^16^, ^18^, ^19^, ^20^]

The incorporation of plant extracts into modified chitosan (CS) films has demonstrated the potential to create materials with enhanced antimicrobial and antioxidant properties, as evidenced by studies. CS films containing oak (*Quercus robur*) and hop (*Humulus lupulus*) extracts exhibited high optical and mechanical properties, good light blockage, and great antimicrobial activity against *B. subtilis*.^[16]^ Deshmukh *et al*. prepared films from CS and defatted *Chlorella* biomass with high mechanical and barrier properties and high biodegradability.^[21]^ The films based on CS and *S. officinalis* and *H. perforatum* extracts can inhibit well the growth of *S. aureus*, *MRSA*, *E. coli*, *P. aeruginosa* bacteria.^[23]^ These results indicate that the development of CS‐based composites incorporating novel plant extracts remains a vibrant area of research.

Among plants with antimicrobial and anti‐inflammatory ability, *Rhodomyrtus tomentosa* (Aiton) Hassk, an herb has been used widely in traditional medicine. The extract from *Rhodomyrtus tomentosa* has demonstrated promising therapeutic potential due to its rich polyphenol content and diverse pharmacological effects.[^24^, ^25^, ^26^, ^27^] This extract effectively inhibits the growth of various bacteria, such as *S. pyogenes*, *S. aureus*, and *E. coli*.[^28^, ^29^, ^30^] Additionally, the extracts exhibit an excellent anti‐propionibacterium acnes activity.[^31^, ^32^] The extract reduces inflammation by modulating the expression of inflammatory mediators and suppressing the activity of inflammatory enzymes,[^33^, ^34^] also scavenges free radicals and protects cells from oxidative damage.[^30^, ^34^, ^35^]

Despite the promising potential of *Rhodomyrtus tomentosa* extract, further research is required to fully understand its mechanism of action, optimize its delivery, and assess its long‐term safety and efficacy. In the report of Senait *et al*., the ethyl acetate extract of *Rhodomyrtus tomentosa* contributed significantly to the increase in the antimicrobial effect of poly(vinyl alcohol) electrospun nanofibers.^[36]^ In another report, the hydrogels based on alginate and *Rhodomyrtus tomentosa* leave extract with great anti‐bacterial, anti‐inflammatory, and cell proliferative activities are potential for applications in wound treatment and tissue regeneration.^[37]^ Therefore, incorporating this extract into a chitosan‐based delivery system could offer a synergistic approach to address the limitations of both materials. This incorporated biocomposite film is expected to have good applications in biomedical such as acne treatment or wound healing.

The solution method is a popular method for the preparation of films based on CS and plant extract.[^9^, ^19^, ^21^, ^22^, ^23^] To overcome challenges related to film thickness uniformity and the potential aggregation of plant extracts during solvent evaporation, 3D printing served as a feasible solution. The 3D‐printing technology has emerged as a powerful tool for fabricating patient‐specific medical devices and biomimetic tissue engineering scaffolds due to its ability to construct complex structures with high precision.[^38^, ^39^, ^40^] The 3D printing techniques have facilitated the fabrication of these complex structures, enabling the controlled loading and releasing procedure of drug delivery systems.[^1^, ^3^, ^40^, ^41^, ^42^] This method has several advantages such as controllable thickness and good uniformity. Additionally, 3D printing enables the precise deposition of gel‐like materials onto diverse substrates including cotton and medical gauze, thereby expanding the scope of their applications.

This work presents the development of a 3D‐printed chitosan film loaded with an enriched phenolic extract from *Rhodomyrtus tomentosa* leaves, aiming at to address the limitations of both pure chitosan and crude plant extracts. By combining chemically modified chitosan with a potent phenolic‐rich extract, the resulting film is expected to exhibit enhanced mechanical properties, antimicrobial, and anti‐inflammatory activities. Besides, the loading capacity and release kinetics of the phenolic compounds have been evaluated and discussed.

## Materials and Methods

### Materials

*Rhodomyrtus tomentosa* leaves were collected in March 2023 from Tam Dao town, Vinh Phuc province, Vietnam. Methanol, ethanol, acetone, n‐hexane, ethyl acetate, and acetic acid, are all sourced from China. Analytical grade reagents including FeCl_3_, gallic acid standard solution, Na_2_CO_3_, and Folin‐Ciocalteu reagent were bought from Sigma‐Aldrich (Massachusetts, United States). Indigo carmine indicator, KMnO_4_, and vanillin reagent were sourced from Vietnam. The Dianion HP‐20 resin (Merck, Germany), and polyamide 6 (Sorbtech, USA) were used for column chromatography, and Silica gel 60 F254 plates (Merck, Germany) were for thin‐layer chromatography (TLC). Chitosan medium molecular weight (viscosity of 200–800 cP), and sodium tripolyphosphate (TPP, 85 %) were purchased from Sigma‐Aldrich. RAW264.7 cells, *E. coli*, and *S. aureus* bacterial strains were provided by ATCC (American Type Culture Collection, Manassas, VA, USA). Luria Bertani broth/Agar (LB), and Mueller Hinton Agar (MHA) were purchased from Himedia, India. Dulbecco's Modified Eagle Medium (DMEM), MTT (3‐(4,5‐dimethylthiazol‐2‐yl)‐2,5‐diphenyltetrazolium bromide), Griess reagent, LPS (lipopolysaccharide) were purchased from Sigma‐Aldrich (Merck KGaA, Darmstadt, Germany) Thermo Fisher Scientific (Waltham, MA, USA).

### Extraction of Polyphenol‐Rich Fractions from *Rhodomyrtus tomentosa* Leaves

500 grams of dried *Rhodomyrtus tomentosa* leaves underwent ultrasonic‐assisted extraction for one h at 50 °C. The solvent system employed consisted of ethanol, water, and acetic acid at a volumetric ratio of 6 : 4:0.1 % (v/v). The resulting extract was filtered and collected for further processing. After that, the filtered extract, amounting to about 1000 mL, was concentrated by rotary evaporation at 50–60 °C under vacuum to remove alcohol, yielding an aqueous extract.

Liquid‐liquid extraction for 500 mL of the aqueous extract was performed 3–4 times using n‐hexane and ethyl acetate to obtain three fractions: n‐hexane extract (SH), ethyl acetate extract (SE), and the remaining aqueous extract (SW).

The presence of acetic acid created an acidic environment with a pH 3, which increased the polarity of the solvent and enhanced the efficiency of the extraction process. The ethanol extract obtained was a brown liquid in appearance. Further purification involved concentrating the ethanol extract to remove the solvent, yielding an aqueous extract. This aqueous extract was then partitioned with n‐hexane to eliminate non‐polar substances and subsequently with ethyl acetate to remove medium polarity substances, resulting in a polar, polyphenol‐rich aqueous extract. Concentration of the aqueous extract under vacuum yielded 224.8 g of aqueous extract residue (SW), which was enriched in polyphenol compounds.

### Column Chromatography

22.4796 g of the aqueous extract residue (SW) were applied to a chromatography column packed with 90 g of Dianion HP‐20 adsorbent with a particle size of 250 μm. A gradient elution was performed using an acetone: water solvent system with increasing polarity (9 : 1, 5 : 1, 3 : 1). The eluates were collected in test tubes (Ø16x160 mm) and analyzed using thin‐layer chromatography (TLC)^[43]^ observed under UV light at a wavelength of 254 nm. Spots were visualized with 5 % vanillin sulfate in alcohol and heated at 120–150 °C for 1–2 min. Similar fractions were combined to yield six distinct fractions, labeled SW1 through SW6. These fractions were then subjected to concentration by rotary evaporation to remove solvents and isolate polyphenols. The SW3 fraction was obtained with a weight of 5.84 g.

**Figure 1:**
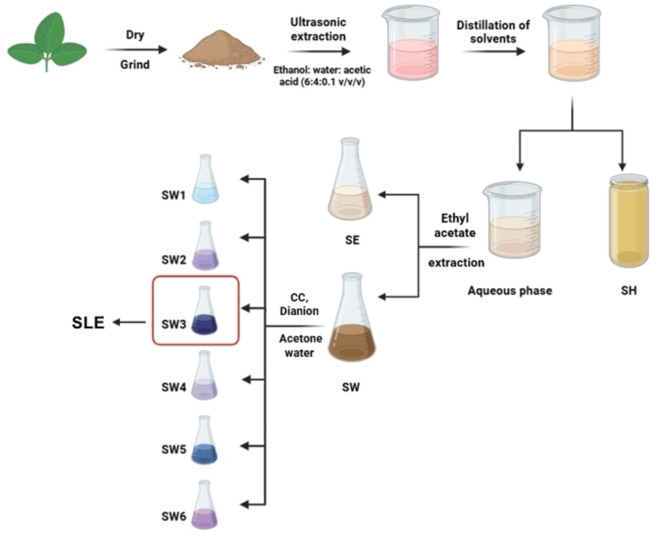
Fractional extraction diagram of polyphenol‐rich fractions for Rhodomyrtus tomentosa leaves.

The qualitative analysis of polyphenols in the crude ethanol extract of *Rhodomyrtus tomentosa* leaves was carried out using two reagents −1 % FeCl_3_ and 0.5 % gelatin/NaCl. For the FeCl_3_ test, two test tubes were prepared ‐ one with 2 mL of the extract as a control, and the other with 2 mL of the extract plus 3–5 drops of the 1 % FeCl_3_ solution. A positive reaction was indicated by a color change from brown to blue‐black in the second test tube. The polyphenol compounds were detected by spraying with 10 % H_2_SO_4_ and 5 % vanillin sulfate, and the plate was observed under UV light at 254 nm. This process allows for the isolation of polyphenol‐rich fractions from *Rhodomyrtus tomentosa* leaves. Figure 1 presents the fractional extraction diagram of polyphenol‐rich fractions for *Rhodomyrtus tomentosa* leaves (SW3, abbreviated as SLE). The TPC content of the obtained SLE was then measured according to the method reported by Makori *et al*.^[44]^

### Preparation of 3D‐Printed CS‐Based Biocomposite Films

#### Preparation of Hydrogel Samples Based on CS and SLE

The procedure for preparation of hydrogel samples based on CS and SLE is as follows: Firstly, 1 g of CS was dissolved in 20 mL of acetic acid 1 % at room temperature for 60 min. Next, TPP solution was added to the CS solution with a ratio of 1.5 wt.% as compared to CS weight. The mixture was stirred in a magnetic stirrer for 3 h at room temperature to cross‐link CS molecules. The SLE was added to the modified CS solution at different concentrations (0.5, 1, 2, and 5 wt.%) to obtain hydrogel samples. The hydrogels were magnetically stirred and homogenized for 3 h to ensure homogeneity, then used as an ink for 3D printing. The hydrogel samples containing 0.5, 1, 2, and 5 wt.% of SLE were abbreviated as mCS0.5PP, mCS1PP, mCS2PP, and mCS5PP, respectively.

#### 3D Printing Process for the Preparation of CS‐Based Biocomposite Films

**Figure 2:**
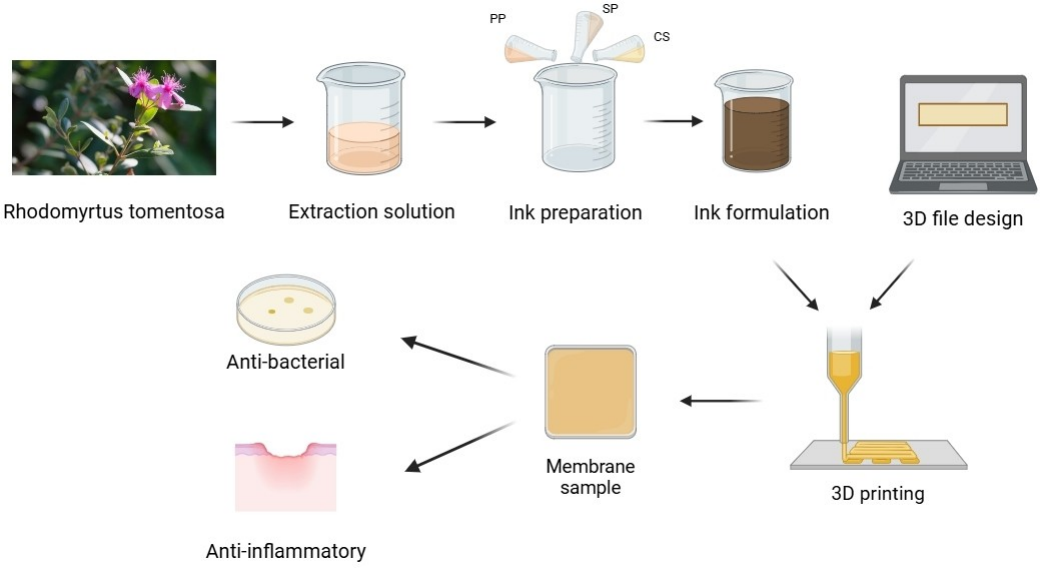
The illustration of the process of preparing chitosan films containing Rhodomyrtus tomentosa leaf extract includes four main parts: (1) Extract generation: Rhodomyrtus tomentosa leaves are extracted to obtain polyphenol‐rich extracts; (2) Ink preparation: Modified chitosan is mixed with the extract at different concentrations; (3) 3D printing file design and 3D printing: Using design software to create a printing pattern, then 3D printing is performed to fabricate chitosan films; (4) Film product evaluation: The printed chitosan films are tested for antibacterial and anti‐inflammatory properties.

Before 3D printing processing, the shape of samples was designed with dimensions of 50×50×0.4 mm on the computer using 3D Builder software. It will be calculated to form G‐code for a 3D printing device by KISSlicer software with the parameters involving the layer thickness of 0.2 mm, extrusion width of 0.6 mm, infill of 100 %, and bed temperature of 60 °C. After calculation, the G‐code file was used to control the 3D printing. The CS/SLE hydrogel samples were input into the syringe of a 3D printing device that was made by the Institute for Tropical Technology, Vietnam Academy of Science and Technology. Next, the 3D printing was run with a printing speed of 10 mm/s. The number of printing layers was varied by 1, 2, and 3 layers to investigate the effect of the printing layer numbers on the properties of biocomposite films. The printed samples were then naturally dried and stored in a PE bag for further characterization. Figure 2 describes the procedure for the preparation of 3D‐printed CS/SLE biocomposite films.

### Characterization

#### Infrared (IR) Spectroscopy

The IR spectroscopy technique was utilized to analyze the CS‐based biocomposite films’ functional groups. The IR spectra of samples were recorded using a Nicolet iS10 spectrometer (USA) with the attenuated total reflection technique in the wavenumber range of 4000–400 cm^−1^ and a resolution of 8 cm^−1^ and 32 scans.

#### Scanning Electron Microscopy (SEM)

SEM images of the CS‐based biocomposite films were taken using a FESEM S4800 device (Hitachi, Japan). The film samples were coated with a gold thin layer to increase the conductivity.

#### Swelling Ability Study

The swelling behavior of the CS‐based biocomposite films was evaluated in simulated body fluid (SBF). The 2 cm×2 cm film specimens were immersed in 100 mL of SBF. At 30‐min intervals, the films were taken, excess surface water was gently removed, and their weights were recorded.^[45]^ The swelling ratio of the samples was monitored continuously for the first 8 h and after 24 h of testing.

#### Drug Release Study

The release of SLE from the CS‐based biocomposite films was tested in SBF at 37 °C for 6 h. The 2 cm×2 cm film specimens were weighed and immersed in 100 mL of SBF. At one‐h intervals, the solution was withdrawn and the fresh SBF was added to the mixture to maintain the volume of samples. The solution was on a Libra S80 UV‐Vis spectrometer (Biochrom, UK) at the λ_max_=261 nm. The content of SLE release from the biocomposite films was calculated according to the following calibration equation: y=1644.7x+0.0507 (R^2^=0.9991).

#### Antibacterial Activity Assay

The antibacterial activity of the *Rhodomyrtus tomentosa* leaf extract (SLE) and CS‐based biocomposite films was evaluated using the agar well diffusion method against *S. aureus* (gram‐positive) and *E. coli* (gram‐negative).[^46^, ^47^] Film samples were cut into a dimension of 1 cm^2^ and placed onto Mueller‐Hinton (MH) agar plates inoculated with bacteria (10^8^ CFU/mL). The plates were maintained at 4 °C for 4 h, then incubated at 37 °C for 24 h. After incubation, the inhibition zones were measured to assess the antibacterial efficacy. Control samples included 0.25 mg/mL ampicillin (positive control), and 70 % ethanol and sterile distilled water (negative control). The minimum inhibitory concentration (MIC) was determined using a modified version of the method described by Mogana *et al*. (2020).^[48]^ The MIC of each sample was identified by serial two‐fold dilutions, with the lowest concentration that exhibited a visible inhibition zone taken as the MIC value.

#### Anti‐Inflammatory Activity Assay

The anti‐inflammatory activity assay involved evaluating the potential of chitosan/SLE film using the RAW264.7 macrophage cell line.[^33^, ^34^] The cells were cultured, and treated with SLE, mCS1PP, mCS, and lipopolysaccharide (LPS) to induce an inflammatory response. The production of nitric oxide (NO), an inflammatory mediator, was measured using the Griess reagent assay. The ability of the membranes to inhibit NO production indicated their anti‐inflammatory efficacy, with IC_50_ values calculated to represent the concentration required to inhibit 50 % of NO production.

#### MTT Assay for Cell Viability

The MTT assay for cell viability assessed the cytotoxicity of the membranes using the RAW264.7 macrophage cell line. Cells were seeded in 96‐well plates and treated with 3 different concentrations (16 μmol/L, 64 μmol/L, 256 μmol/L) of SLE, mCS1PP, and mCS. Cardamonin with 0.3 μmol/L and 3.0 μmol/L are used as positive control. After 24 h of incubation, the MTT reagent was added, and the cells were incubated to form formazan crystals. The crystals were dissolved in DMSO, and absorbance was measured at 570 nm using a microplate reader. Cell viability percentages were calculated by comparing treated samples to control, providing insights into the biocompatibility of the membranes.

#### Data Analysis

Statistical analysis was performed using Origin 2021 software. Results were expressed as mean±standard deviation. Differences between groups were analyzed using one‐way ANOVA followed by Tukey's post hoc test. A p‐value <0.05 was considered statistically significant.

## 2 Results and Discussion

### 2.1 Total Polyphenol Content and Antibacterial Ability of Enriched Polyphenol Extraction from *Rhodomyrtus tomentosa* Leaves

The objective of analyzing the polyphenol content in extract fractions was to identify which fractions are richest in polyphenols for potential applications. The SW fraction contained 37.771 mgGAE/g of polyphenols with 12.25 % tannin. Using dianion chromatography, six fractions (SW1–SW6) were obtained, among them, the SW3 is the richest in polyphenols, exhibiting a total polyphenol content (TPC) of 55.326 mgGAE/g and a tannin content of 19.20 %. This suggests that the enrichment of polyphenols and tannins in the extract is necessary to enhance the content of these compounds in the extract. In a study by Idris *et al*., the highest value of total phenolic extraction from *Rhodomyrtus tomentosa* leaves is 191.97±0.19 mg GAE/g in the case of using 5.0 kg of dried *Rhodomyrtus tomentosa* leaves.^[49]^ So, for 500 g of dried leaves of *Rhodomyrtus tomentosa*, the obtained TPC value is quite high. This is thanks to the assistance of ultrasonication in the extraction process, which helps to extract more organic compounds from herbs.^[50]^ The SW3 fraction or SLE is particularly rich in polyphenols, therefore, it has been selected for evaluating its antimicrobial ability.

**Figure 3:**
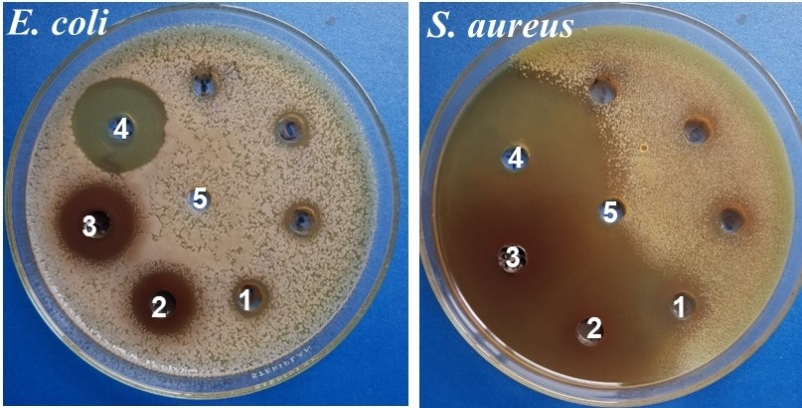
Images of agar plates of antibacterial test of the extract samples. (1) Total ethanol extract, (2) SW fraction, (3) SW3 fraction (SLE), (4) Positive control, and (5) Negative control.

Table 1 and Figure 3 show the antibacterial assessment involving the tests of the total ethanol extract, SW, and SW3 fractions against *E. coli* and *S. aureus*. It can be seen that all tested samples can inhibit the growth of *E. coli* and *S. aureus*, however, their antibacterial capacity is significantly different. The total ethanol extract exhibited antibacterial activity against both *E. coli* and *S. aureus* with an inhibition zone of 5 and 16 mm, respectively. The SW and SW3 fractions showed excellent antibacterial activity with the inhibition zone for *E. coli* ranging from 15–19 mm and the inhibition zone for *S. aureus* above 30 mm. The SW3 exhibited higher antibacterial activity than SW. The enrichment of polyphenols and tannins contributed remarkably to improving the SW3 fraction because polyphenols and tannins have been known as effective antibacterial agents.[^51^, ^52^, ^53^] They can activate to kill bacteria directly or inhibit their growth depending on the type of bacteria that they contact. Some antibacterial mechanisms of polyphenols and tannins can be guested as follows: (1) disruption of cell membrane integrity, (2) protein binding, (3) inhibition of biofilm formation, (4) generation of reactive oxygen species (ROS), (5) inhibition of enzyme activity, (6) interference with DNA and RNA synthesis, (7) metal ion interaction.^[51]^

**Table 1:** Antibacterial activity of the extract from dried Rhodomyrtus tomentosa leaves.

No.	Sample	Zone of inhibition diameter (D‐d) mm	
		*E. coli*	*S. aureus*
1	Total ethanol extract	5	16
2	SW	15	>30
3	SW3 (SLE)	19	>30
4	Positive control	22	44
5	Negative control	0	0

Table 2 presents the antibacterial activity of SLE at different diluting ratios (the initial concentration of SLE is 0.686 g/mL). The SLE can exhibit a good inhibition on the growth of *S. aureus* after diluting to 200 times (3.430 mg/mL) while it only inhibits the growth of *E. coli* after diluting to 80 times (8.570 mg/mL). This suggests that SLE exhibits a stronger inhibitory effect on the growth of *S. aureus* compared to *E. coli*. The differential inhibitory effects of SLE on these two types of bacteria can be attributed to several factors. Firstly, *S. aureus* is a Gram‐positive bacterium, while *E. coli* is a Gram‐negative bacterium. The cell wall of Gram‐positive bacteria consists of a thick peptidoglycan layer, which can be more susceptible to tannins or polyphenols that interact with the cell wall components.^[53]^ Conversely, the outer membrane of Gram‐negative bacteria like *E. coli* acts as a barrier that can limit the penetration of tannins, and polyphenols, making them less effective.

**Table 2:** Antibacterial activity of SLE at different dilution levels.

SLE concentration (mg/mL)	Zone of inhibition diameter (D‐d) mm			
	After 24 h		After 96 h	
	*E. coli*	*S. aureus*	*E. coli*	*S. aureus*
3.430	0	16	0	0
4.288	0	24	0	0
8.570	3	30	3	14
17.150	6	40	6	30
34.300	9	46	9	40
68.600	13	54	13	40
Positive control (0.25 mg/mL)	34	54	34	46
Negative control	0	0	0	0

As observed from Table 2, after 96 h of culturing at 37 °C, the antibacterial activity of SLE against *E. coli* was still maintained while that against *S. aureus* slightly decreased. *S. aureus* tends to regrow in case of using low concentration of SLE. The MIC values of SLE for *E. coli* and *S. aureus* were determined to be 8.570 mg/mL and 3.430 mg/mL. These results suggest that SLE possesses significant antibacterial properties, particularly against *S. aureus* and *E. coli*. However, the slight decrease in its antibacterial ability over a long period could limit the application ability of SLE in practice. Thus, this extract is combined with CS to increase and maintain antibacterial effectiveness for a longer time.

### 2.2 Influence of Printing Layer Numbers on the Morphology and Swelling Degree of 3D‐Printed CS/SLE Biocomposite Films

**Figure 4:**
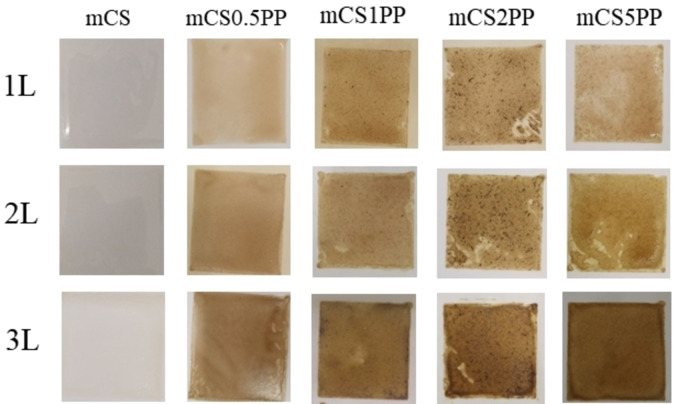
Naked‐eye images of 3D‐printed CS/SLE biocomposite films.

The printing process involved preparing films with single (1 L), double (2 L), and triple (3 L) layers for each composition to evaluate the consistency and quality of the 3D‐printed CS‐based biocomposite films. The naked‐eye images of the CS/SLE biocomposite films are shown in Figure 4. It is seen that the number of printing layers affects the morphology of the final films. In the case of the samples prepared with one printing layer, their surface is uneven. In contrast, for the samples prepared with two printing layers, the obtained films are uniform. However, the SLE agglomerated in the CS matrix in the samples prepared with three printing layers. This suggests that two printing layers are suitable for the preparation of 3D‐printed CS/SLE biocomposite films.

From Figure 4, it is observed that with the increasing SLE concentration in the films, the agglomeration of SLE becomes clearer. This will be discussed in more detail in the 3.3 subsection.

**Figure 5:**
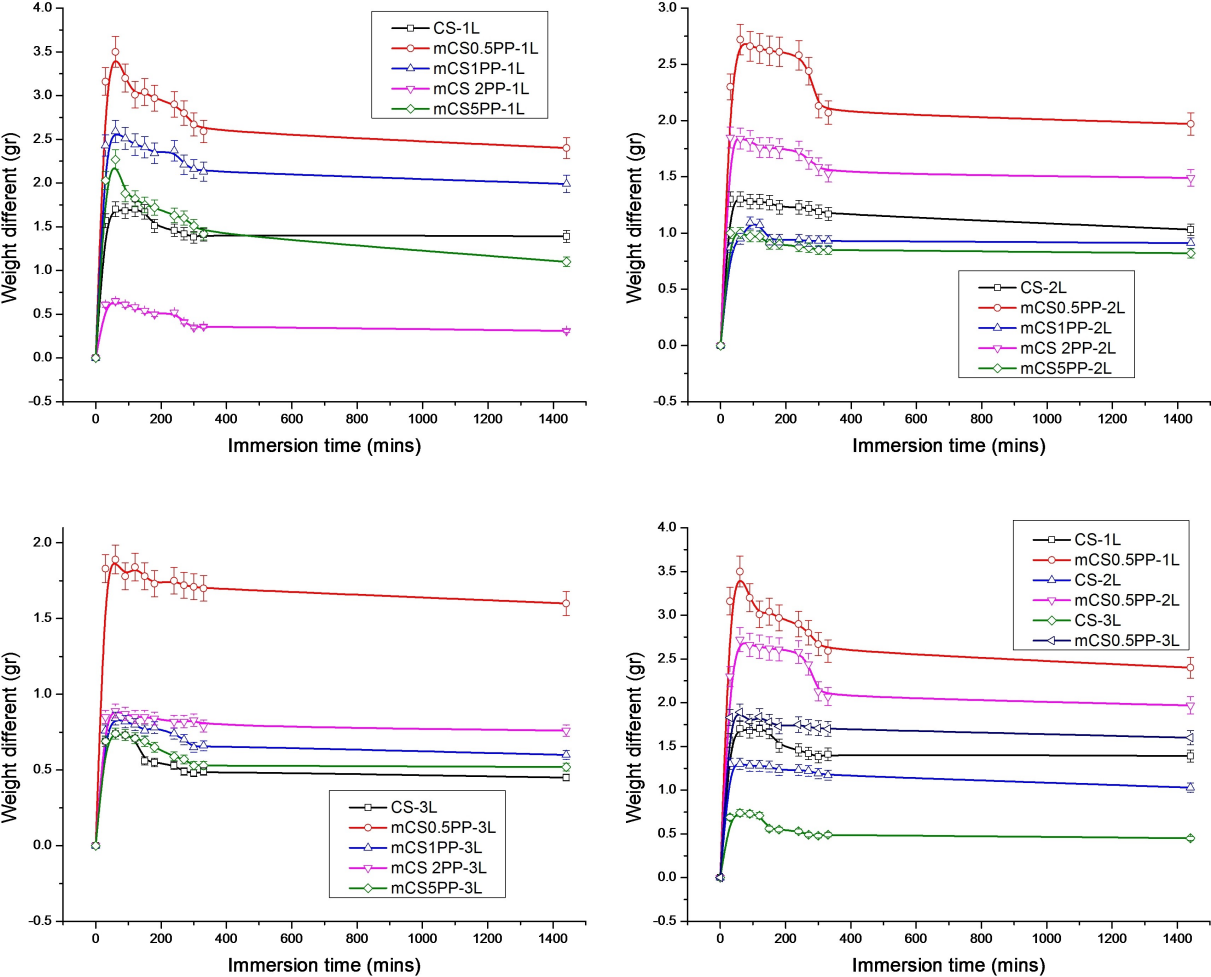
Swelling ability of the 3D‐printed CS/SLE biocomposite films by layer thickness (1 L, 2 L, 3 L) over time.

The swelling behavior in simulated body fluid (SBF) is an important parameter for applications of biocomposite films in wound healing or acne treatment because this will reflect the fluid adsorption ability of the films when they contact with the acne area or scratch. Moreover, the swelling ability has been also used to evaluate the biocompatibility of the biocomposite films.[^54^, ^55^, ^56^] Thus, the effect of printing layer numbers on the swelling behavior of 3D‐printed CS/SLE biocomposite films was assessed and presented in Figure 5. The 3D printing technology also contributed to the preparation of the films with porous structures, leading to these films being swollen. According to Figure 5, the degree of swelling was observed to decrease with increasing films’ thickness (increasing the number of printing layers). This observation can be explained by the denser and more tightly packed structure of the double‐layer and triple‐layer films compared to the single‐layer film. The increased density reduces the available space for water uptake, leading to lower swelling. The good swelling ability of the biocomposite films suggests good biocompatibility.

The influence of SLE concentration on the swelling ability of the biocomposite films is also exhibited in Figure 5. While a clear trend was not observed, the presence of SLE generally appeared to affect the swelling behavior of the films. This effect could be attributed to interactions between the SLE components and the CS matrix, potentially altering the water absorption capacity of the material.

Regarding the effect of immersion time, the swelling of the films initially increased with time up to 60 min. This initial increase can be attributed to the gradual penetration of water molecules into the CS matrix. However, after 60 min, a slight decrease in swelling was observed. This decrease could be due to the release of organic components such as phenolic compounds from the films into the SBF, leading to a reduction in their water‐absorbing capacity. Additionally, prolonged immersion may induce structural rearrangements within the CS matrix, potentially affecting its swelling behavior.

After 24 h of immersion, a significant decrease in swelling was observed for all samples. This sustained reduction in swelling could be explained by the combined effects of organic compound release, structural rearrangements, and potential degradation of the chitosan matrix over extended immersion periods.

Combining naked‐eye images with the swelling results, the suitable condition for the preparation of 3D‐printed CS/SLE biocomposite films is two printing layers. Based on that, the structure, morphology, drug release content, drug release kinetic as well as bioactivities of these films will be assessed and discussed.

### 2.3 Structure, Morphology, Mechanical Properties, and Drug Release Content from 3D‐Printed CS/SLE Biocomposite Films

**Figure 6:**
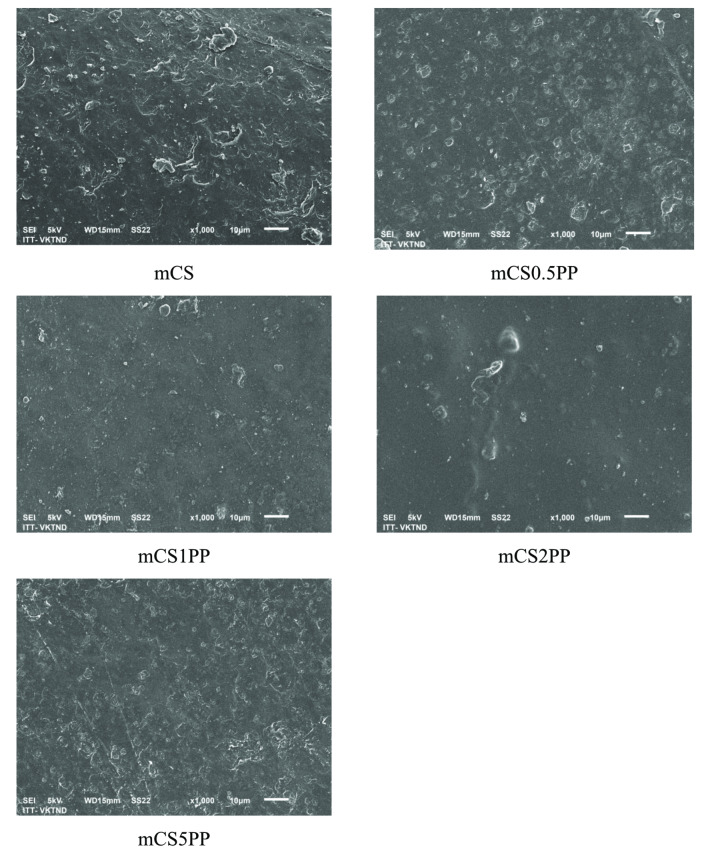
SEM images of 3D‐printed CS/SLE biocomposite films (x1000).

Figure 6 presents SEM images of 3D‐printed CS/SLE biocomposite films prepared with two printing layers at a magnification of 1000x. All films reveal a heterogeneous and dense structure with a continuous phase. The SLE was uniform distribution within the CS matrix, particularly in the mCS1PP sample. This is attributed to the presence of hydroxyl (−OH), carboxyl (−COOH), and amine (−NH_2_) functional groups in the SLE and CS, facilitating hydrogen bonding and electrostatic interactions between CS and SLE.[^18^, ^19^, ^20^] When increasing the SLE content in the films, the structures of the films become uneven due to the agglomeration of SLE as shown in Figure 4 before.

**Figure 7:**
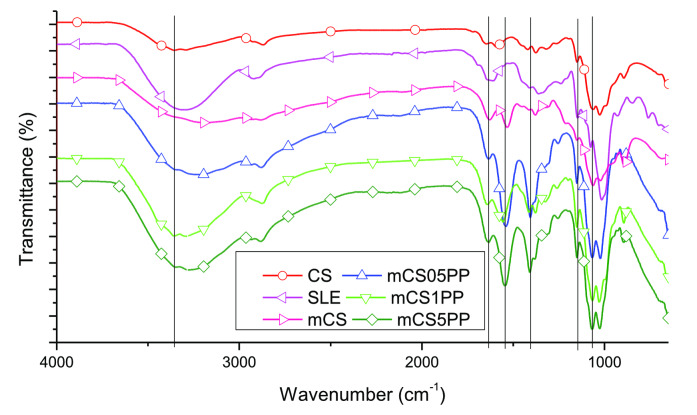
IR spectra of SLE, CS, mCS, and 3D‐printed CS/SLE biocomposite films.

Figure 7 reveals the IR spectra of the CS, modified CS (mCS), SLE, and 3D‐printed CS/SLE biocomposite films.

The IR spectrum of the SLE exhibits distinct peaks at the wavenumbers of 3400, 2928, 1720, 1605, 1446, 1333, 1192, 1032, and 644 cm^−1^, corresponding to O−H, C−H, C=O, C=C, C−C, C−O functional groups in the polyphenols, tannins, and other organic compounds in the extract.^[20]^ For CS, mCS, and CS/SLE films, key functional group peaks were identified in their IR spectra, including 3254 cm^−1^ for the stretching vibration of O−H groups, 2928 cm^−1^ for stretching vibration of C−H groups, 1720 cm^−1^ for C=O stretching (amide I), 1605 cm^−1^ for O−H bending and C=C−C bond stretching, 1556 cm^−1^ for N−H bending, 1446 cm^−1^ for aromatic C−H stretching, 1333 cm^−1^ for C−N bending, and 1020–1192 cm^−1^ for C−O, C−C stretching.[^10^, ^16^, ^44^, ^45^] These peaks confirmed the incorporation of polyphenol components into the CS matrix, validating the successful modification of the membranes. The concentration of SLE in the biocomposite films causes a negligible effect on the vibrations of functional groups of these films.

**Figure 8:**
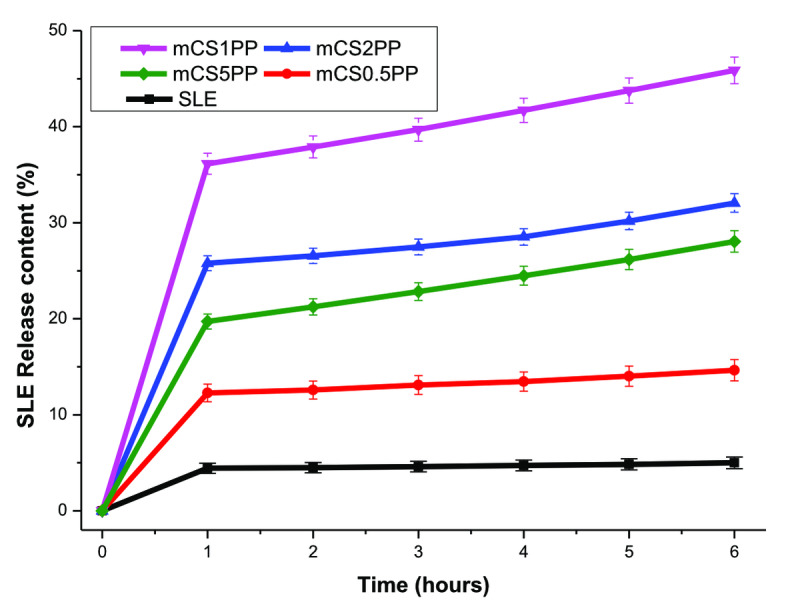
SLE release content from the SLE (unloaded) and 3D‐printed CS/SLE biocomposite films in SBF.

The SLE release content from the SLE unloaded in the CS matrix and from the 3D‐printed CS/SLE biocomposite films in SBF is shown in Figure 8. It is seen that SLE has poor solubility in SBF with the SLE release content reaching only 5 % after 6 h of testing. Consequently, the SLE can be well released from the 3D‐printed CS/SLE biocomposite films, with the SLE content reaching 14.64–45.86 % depending on the SLE concentration. The SLE was released continuously from the films during the test, indicating a controllable release for SLE when loaded in the modified CS matrix. As compared to the chitosan/alginate/*Camellia chrysantha* polyphenols,^[57]^ the SLE release content in this study is lower. This may be due to the difference in the polyphenols content in the extracts as well as the nature of polymer‐carriers. Among tested film samples, the mCS1PP sample released the highest SLE content in SBF. This is thanks to the uniformity in the morphology of mCS1PP as well as the great dispersity of SLE in the CS matrix at the content of 1 wt.%. Moreover, the adequate swelling ability of mCS1PP also contributed to the control of the SLE release from the composite films.

The Zero‐order kinetic, First‐order kinetic, Higuchi model, Hixson‐Crowell model, and Korsmeyer‐ Peppas model have been used for assessing the drug release mechanism from the 3D‐printed CS/SLE biocomposite films in SBF.[^57^, ^58^] Based on the regression coefficient values (Table 3), the Korsmeyer‐ Peppas model is the most suitable for describing the SLE release from the biocomposite films. This is in agreement with the reports by Luong and Pravilović,[^57^, ^58^] in which, the SLE release process is complex with anomalous diffusion of polyphenols controlled by molecular diffusion and polymer relaxation.

**Table 3:** Regression coefficients (R^2^) of kinetic models reflecting drug release mechanisms of 3D‐printed CS/SLE biocomposite films in SBF.

Sample	Zero‐order kinetic	First‐order kinetic	Higuchi model	Hixson‐ Crowell model	Korsmeyer‐ Peppas model
SLE	0.9820	0.9861	0.9361	0.9848	0.9861
mCS0.5PP	0.9881	0.9924	0.9499	0.9911	0.9924
mCS1PP	0.9986	0.9999	0.9742	0.9997	0.9999
mCS2PP	0.9678	0.9778	0.9132	0.9747	0.9778
mCS5PP	0.9988	0.9999	0.9785	0.9997	0.9999

### 2.4 Antibacterial and Anti‐Inflammatory Activities of 3D‐Printed CS/SLE Biocomposite Films

**Figure 9:**
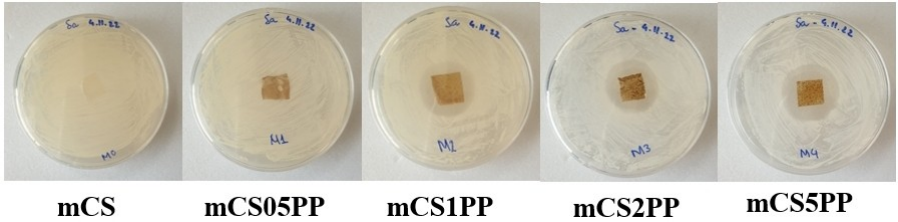
The inhibition zone of the CS/SLE biocomposite films against *E. coli*.

The antibacterial activity of the 3D‐printed CS/SLE biocomposite films against *E. coli* and *S. aureus* is presented in Table 4. The mCS sample showed no inhibition zone, indicating no antibacterial activity. Similarly, the mCS0.5PP sample exhibited no inhibition zone. This is maybe due to the low concentration of SLE in the sample, making it only inhibit the bacteria at the contact position but cannot diffuse to the agar environment to kill the bacteria. In contrast, the mCS1PP sample produced a 20 mm zone of inhibition for *E. coli* and 16 mm one for *S. aureus*, the mCS2PP sample produced 18 mm and 16 mm zones of inhibition for *E. coli* and *S. aureus*, and the mCS5PP sample produced 22 mm and 18 mm zones of inhibition for *E. coli* and *S. aureus*, respectively. These results are visually represented in the images included in Figure 9, which show the antibacterial activity assay against *E. coli* for the different CS/SLE biocomposite films. The chitosan/SLE films exhibit antibacterial activity against *E. coli* better than that against *S. aureus*, similar to the report of Gradinaru *et al*. for chitosan/plant extract membranes.^[23]^ The samples containing SLE (1–5 wt.%) exhibited significant antibacterial activity, highlighting the role of SLE in enhancing the antibacterial properties of the biocomposite films. This finding aligns with previous studies that have demonstrated the antimicrobial potential of *Rhodomyrtus tomentosa* polyphenols.[^27^, ^28^, ^29^, ^30^, ^51^] The incorporation of SLE into the chitosan matrix significantly improved its antibacterial efficacy, making these films promising candidates for biomedical applications where antibacterial properties are crucial. The mechanisms involving the antimicrobial activity of chitosan‐based biofilms have been summarized by Khubiev *et al*.,^[59]^ including (i) cell membrane disruption, (ii) chelation of nutrients, (iii) interference with microbial gene expression, and (iv) reactive oxygen species generation.

**Table 4:** Antibacterial activity of mCS and mCS/PP samples after 24 h incubation.

No.	Sample	Zone of inhibition diameter (D‐d) (mm)	
		*E. coli*	*S. aureus*
1	mCS	0	0
2	mCS0.5PP	0	0
3	mCS1PP	20	16
4	mCS2PP	18	16
5	mCS5PP	22	18
6	Positive control	24	26
7	Negative control	0	0

One of the objectives of this study is to demonstrate the anti‐inflammatory capability of the films in the case of the combination of CS and SLE. The results presented in Table 5 detail the nitric oxide (NO) inhibition capabilities of various test samples at different concentrations. At a concentration of 256 μg/L, the SLE sample inhibited NO production by 68.26±0.86 %. At 64 μmol/L, the inhibition was 55.09±0.27 %, and at 16 μg/L, it was 48.03±0.81 %. These results indicate that SLE has a dose‐dependent NO inhibition capacity, with effectiveness decreasing at lower concentrations. This result is similar to the reports of Ontong and Jeong.[^24^, ^25^] For the mCS1PP sample, at 256 μg/L, the NO inhibition was 77.70±0.21 % and it decreased to 49.09±0.15 % at the lowest concentration. This data demonstrates that mCS1PP exhibits better NO inhibition compared to SLE, indicating that the combination of chitosan and polyphenol significantly enhances anti‐inflammatory efficacy. Similarly, the mCS sample showed NO inhibition of 76.01±0.43 % at 256 μg/L, 57.00±0.27 % at 64 μg/L, and 47.69±0.15 % at 16 μg/L. It also suggests that mCS also has a high NO inhibition capacity. These results suggest that incorporating SLE into the CS significantly enhances its anti‐inflammatory efficacy.

**Table 5:** Anti‐inflammatory activity and cellular toxicity of SLE and 3D‐printed CS/SLE biocomposite films.

Sample name	Concentration (μg/L)	NO inhibition (%)	Cell viability (%)	IC50 value (μg/mL)
Negative control (−)	–	100.0±1.3	104.76±0.15	
Positive control (+) [Cardamonin]	0.3	45.85±2.12	86.47±0.21	
	3.0	86.93±0.96	71.8±0.51	0.61
LPS	–	0.0±0.9	100.0±0.13	
SLE	256	68.26±0.86	61.39±0.38	22.76
	64	55.09±0.27	102.13±0.13	
	16	48.03±0.81	112.56±0.26	
mCS1PP	256	77.70±0.21	52.02±0.11	19.40
	64	57.07±0.29	100.31±0.29	
	16	49.09±0.15	103.13±0.17	
mCS	256	76.01±0.43	50.15±0.12	21.08
	64	57.00±0.27	99.32±0.39	
	16	47.69±0.15	101.14±0.15	

Furthermore, the cell viability results indicate that the tested samples exhibit low cytotoxicity. Compared to the positive and negative controls, the SLE, mCS1PP, and mCS samples did not demonstrate significant cytotoxic effects at lower concentrations. The cell viability rates for the samples at 64 μg/L and 16 μg/L were above 100 %, indicating that the cells continued to proliferate well in the presence of the samples in the culture medium. However, at the high concentration of 256 μg/L, cell viability significantly decreased, particularly for mCS1PP and mCS, with results of 52.02±0.11 % and 50.15±0.12 %, respectively. The IC_50_ values for the SLE, mCS1PP, and mCS samples were 22.76 μg/mL, 19.40 μg/mL, and 21.08 μg/mL, respectively. These findings suggest that while the samples are non‐toxic at lower concentrations, higher concentrations exhibit cytotoxic effects, particularly for the modified chitosan samples. All tested samples exhibited anti‐inflammatory activity, with the mCS1PP sample containing 1 % SLE displaying the strongest effect. This suggests that incorporating SLE into the chitosan matrix significantly enhances its anti‐inflammatory efficacy. The CS facilitates the sustained release and interaction of bioactive components, contributing to the observed synergistic effect.

The results in Table 6 indicate that most reported CS/plant extract films prepared by solution casting method with great antimicrobial activity.[^11^, ^19^, ^56^, ^57^, ^58^, ^59^, ^60^, ^61^, ^62^] However, the anti‐inflammatory activity of these films is still limited in assessment. Comparing the inhibition zone values of reported CS/plant extract films shows strong antibacterial activity of the CS/*R. tomentosa* leaf extract film in this study.

**Table 6:** Comparison the antibacterial and anti‐inflammatory activity of mCS1PP with other reports.

Sample	Preparation method	IC_50_ (NO inhibition) (μg/mL)	Zone of inhibition (mm)	Refs.
CS/oak extract film	Solution casting	–	*E. coli: contact inhibition (+), inhibition zone (−)* *B. subtilis: contact inhibition (+), inhibition zone (+)*	^[16]^
CS/hop extract film	Solution casting	–	*E. coli: contact inhibition (+), inhibition zone (−)* *B. subtilis: contact inhibition (+), inhibition zone (+)*	^[16]^
CS/defatted Chlorella biomass film	Solution casting	–	–	^[21]^
CS/α‐tocopherol film	*In‐situ* crosslinked solution casting	–	–	^[22]^
CS/*S. Officinalis* extract membrane	Impregnation (Absorption of plant solutions)	–	*S. aureus*: 10.16–12.06 *MRSA*: 8.9–11.36 *E. coli*: 9.23–13.23 *P. aeruginosa*: 12.2–12.06	^[23]^
CS/*H. perforatum* extract membrane	Impregnation (Absorption of plant solutions)	–	*S. aureus*: 7.5–8 *MRSA*: 7–9 *E. coli*: 10–12.33 *P. aeruginosa*: 8.2–10.16	^[23]^
CS/collagen hydrolysate/red cabbage extract film	Solution casting	–	*E. coli*: 9.53–10.20 *S. aureus*: 10.52–12.17	^[60]^
CS/aqueous sage and rosemary extract film	Solution casting with ultrasonic assistance	–	*E. coli* *S. aureus* (~4 log CFU/g sample)	^[61]^
CS/poly(vinyl alcohol)/*Phyllanthus reticulatus* fruit extract film	Solution casting	–	*E. coli*: 13.3 *S. aureus*: 12 *A. niger*: 12.68	^[62]^
CS/green tea extract film	Solution casting	–	*Murine norovirus*: 2.5–4 logs *E. coli*, *L. innocua*: undetectable after 24 h exposure	^[63]^
CS/green pea (*Pisum sativum* L.) pod extract gel film	Film‐forming solution	–	*S. typhimurium*: 7.89–15.66 *E. coli*: 8.12–16.25 *B. subtillis*: 10.67–19.42 *P. aeruginosa*: 10.35–18.98	^[64]^
CS/*P. terebinthus* leaf, stem, seed extract film	Solution casting	–	*P. microbilis*, *P. vulgaris*, *P. aeruginosa, E. coli: 28.19–21.33*	^[65]^
CS/polyvinylpyrrolidone/*Eucalyptus citriodora* ethyl acetate‐extract film	Solution casting with ultrasonic assistance	–	*E. coli: 23* *S. aureus: 18* *B. subtilis: 21* *C. albicans: 16*	^[66]^
CS/polyvinylpyrrolidone/ *Eucalyptus citriodora* hexane‐extract film	Solution casting with ultrasonic assistance	–	*E. coli:25* *S. aureus: 21* *B. subtilis: 23* *C. albicans: 19*	^[66]^
CS/polyvinylpyrrolidone/*Eucalyptus citriodora* methanol‐extract film	Solution casting with ultrasonic assistance	–	*E. coli:28* *S. aureus: 17* *B. subtilis: 19* *C. albicans: 15*	^[66]^
CS/*R. tomentosa* leaf extract film	3D printing	19.40	*E. coli*: 18–22 *S. aureus*: 16–18	This study

These findings from the antibacterial and anti‐inflammatory tests support the potential application of CS/SLE film in biomedical fields. The significant anti‐inflammatory effects and antibacterial activity against *S. aureus* and *E. coli* highlight their therapeutic potential in managing inflammatory conditions and infections.

## 3 Conclusions

This study successfully developed a novel 3D‐printed chitosan (CS)‐based biocomposite film enriched with *Rhodomyrtus tomentosa* leaf extract (SLE), demonstrating enhanced antibacterial and anti‐inflammatory properties. The two printing layers were the most suitable for the preparation of the CS/SLE biocomposite films. The 3D printing technique was suitable for producing the CS biocomposite films containing 1 wt.% of SLE with an even structure, adequate swelling degree, and good biocompatibility. The SLE can interact with CS through physical interactions like hydrogen bonds and dipolar‐dipolar interaction. The 3D‐printed CS/SLE biocomposite films had excellent antibacterial activity against *S. aureus* and *E. coli*. Moreover, they also exhibited potent anti‐inflammatory activity with IC_50_ values of 19.40 μg/mL. Specifically, these films showed significant anti‐inflammatory effects with up to 76.01±0.43 % NO inhibition. They also mitigated the detrimental effects of inflammation on cellular viability. These findings suggest that 3D‐printed CS/SLE biocomposite films are promising anti‐inflammatory agents for various biomedical applications, particularly in the treatment of inflammatory skin conditions and infections. Further investigations are warranted to explore their biodegradability and *in vivo* efficacy for specific applications.

## Funding

This work was financially supported by Vietnam Academy of Science and Technology (project code: VAST03.03/22‐23).

## Institutional Review Board Statement

Not applicable.

## Informed Consent Statement

Not applicable.

## Author Contributions

Quan Vo−An: Writing‐original draft, Project administration, Methodology, Funding acquisition. Chinh Thuy Nguyen: Investigation, Formal analysis, Writing‐original draft. Uyen Thu Pham: Writing‐original draft. Tuan Anh Nguyen: Writing‐review and editing. Duong Thanh Nguyen: Data curation, validation. Dung Tran Hoang: Investigation, Methodology. Lu Trong Le: Writing‐review and editing. Quyen Thi Cam Ngo: Validation. Hoang Thai: Supervision, Writing‐review and editing.

## Conflict of Interests

The authors declare no conflict of interest.

4

## Data Availability

Data sharing is not applicable to this article as no datasets were generated or analyzed during the current study. The data that support the findings of this study are available from the corresponding author upon reasonable request.
